# Molecular mechanisms contributing to glucocorticoid resistance in lymphoid malignancies

**DOI:** 10.20517/cdr.2019.29

**Published:** 2019-09-19

**Authors:** Blanca Scheijen

**Affiliations:** Department of Pathology, Radboud University Medical Center and Radboud Institute for Molecular Life Sciences, GA 6525, Nijmegen, the Netherlands.

**Keywords:** Glucocorticoid receptor, therapy resistance, acute lymphoblastic leukemia, non-Hodgkin lymphoma, multiple myeloma

## Abstract

Despite the introduction of many novel therapies into the clinic to target hematological malignancies, glucocorticoids (GCs) still remain one of the cornerstone drugs in first-line treatment of lymphoid tumors. However, a significant portion of the patients display acquired GC therapy resistance. This review will describe the different molecular mechanisms that contribute to GC resistance in lymphoid tumors. These include suppression of glucocorticoid receptor (GR) expression, activation of cell signaling pathways that modulate GR function, differential recruitment of transcriptional co-regulators, and changes in chromatin accessibility. Many of these mechanisms are interconnected to genetic alterations associated with relapsed disease in lymphoid malignancies.

## Introduction

Glucocorticoids (GCs) are primary stress hormones that maintain homeostasis and control a broad range of physiological processes, including immune system function, skeletal growth, reproduction, metabolism, energy production and central nervous system function^[1]^. Based on their strong anti-inflammatory and immune-suppressive actions, synthetic GCs are widely used for the treatment of inflammatory conditions, such as organ transplant rejection, autoimmune diseases and asthma, as well as anticancer therapy for lymphoid malignancies^[2]^. Because of their hydrophobic nature, GCs readily diffuse from the circulation into tissues and cells, where they orchestrate various cellular responses, including cell proliferation, differentiation and apoptosis in a tissue-, cell type- and developmental stage-specific manner. Both endogenous and synthetic GCs exert their action by binding and activating the glucocorticoid receptor (GR) encoded by the *NR3C1* gene, which is a member of the nuclear receptor superfamily of ligand-dependent transcription factors^[3]^. Consistent with the pleiotropic effects of GCs, GR is ubiquitously expressed and necessary for life after birth^[4]^.

Extensive research on the mechanism of action of GCs and GR protein function in mediating sensitivity and specificity of the different biological responses, has made an important contribution in unraveling the complexity of GC signaling and its regulation. Especially, studies directed towards the identification of molecular pathways underlying GC resistance in hematological cancers have yielded valuable information for novel therapeutic approaches to target this clinical problem. This review will focus on the therapeutic effects of synthetic GCs in the treatment of hematological malignancies of lymphoid origin, and mechanisms of acquired resistance to GCs.

## Therapeutic effects of GCs in lymphoid malignancies

The main effects of GCs in lymphoid tissue are growth arrest and induction of programmed cell death. After the initial observation in 1944 that hydroxyl-corticosterone induced apoptosis in malignant mouse lymphocytes, synthetic GCs were implemented in the clinical treatment of lymphomas and leukemia^[5]^. Nowadays, prednisone and dexamethasone still are central drugs in the main therapy of most lymphoid malignancies, both for pediatric and adult patients^[6]^. The cytotoxic effects of GCs have been successful exploited in the cancer therapy of acute lymphoblastic leukemia (ALL), but resistance to GCs *in vitro* and *in vivo* has been imminently recognized as an adverse prognostic factor in ALL^[7]^. ALL is characterized by an accumulation of early immature lymphocytes within the bone marrow compartment and is largely a childhood cancer. B-cell precursor ALL (BCP-ALL) is the predominant subtype (85%), and corticosteroids in conjunction with other chemotherapeutic drugs, such as vincristine, anthracyclines, antimetabolites and asparaginase, together with improved risk stratification have resulted in cure rates exceeding 90% in children^[8]^. In childhood ALL, the prednisone response after 7-day of monotherapy is a prognostic factor utilized in the adaptation of chemotherapy protocols^[9-11]^. In adult ALL, treatments have been adapted from the successful childhood ALL therapy regimens, but response rates are unfortunately less favorable with only 40% disease-free survival after 5 years^[12]^. In part, this is due to the fact that adults tolerate less the intensive chemotherapy regimens, which require adaptation of treatment schedules, and the occurrence of a larger proportion of high-risk ALL subtypes in adult BCP-ALL, such as *BCR-ABL1*-positive ALL. High-risk patients and refractory/relapsed disease will often undergo allogeneic stem cell transplantation (SCT). Novel targeted therapeutic modalities involving tyrosine kinase inhibitors and CD19 chimeric antigen receptor (CAR) T-cell immunotherapy seem to yield more promising results in adult ALL treatment^[13]^.

Chronic lymphocytic leukemia (CLL) is a disease of mature B cells that accumulate over time in the blood stream, lymph nodes and bone marrow. Several studies performed in the 1980s and 1990s showed no additional benefit of corticosteroids to other (mono) therapies and reported severe toxicity, such as myelosuppression and serious infections^[14]^. Therefore, the standard of first-line treatment in CLL consists of immuno-chemotherapy, often fludarabine, cyclophosphamide and anti-CD20 antibody rituximab (FCR), without synthetic GCs. However, high-dose methylprednisolone (HDMP) has been shown to induce apoptosis in cultured CLL cells from patients with relapsed or resistant disease^[15]^, and significant response rates have been observed for HDMP together with rituximab in refractory/relapsed CLL patients^[16-19]^. HDMP has been shown to suppress Wnt signaling in CLL by down-regulating LEF-1 protein expression^[20]^, which acts as a survival factor in CLL^[21]^. Thus, HDMP in conjunction with conventional immuno (-chemo)therapy is one of the options to be considered for high-risk CLL treatment.

Hodgkin lymphoma (HL) shows a bimodal age distribution (15-35 years of age and after 55 years of age), and can be divided in two categories, classic HL (cHL) and the rare entity of nodular lymphocyte-predominant HL^[22]^. cHL is characterized by the presence of CD30-positive multinucleated B-lymphocytes, termed Hodgkin Reed-Sternberg cells, which orderly spread as malignant lymphocytes throughout the lymphatic system. The HRS cells account only for 1%-10% of the tumor tissue in a background of reactive immune cells, creating a highly abundant inflammatory tumor microenvironment^[22]^. Current first-line regimen for patients with HL is mostly ABVD (adriamycin, bleomycin, vinblastine and dacarbazine), sometimes in combination with radiotherapy, which achieves a 5-year progression-free survival of about 80% for advanced stage disease^[23]^. Another protocol, called escalated BEACOPP (bleomycin, etoposide, adriamycin, cyclophosphamide, vincristine, procarbazine and prednisone), shows in some studies a better progression-free survival, but also more side effects (higher mortality rate, secondary malignancies and sterility risk)^[23]^. However, the additive beneficial effects of corticosteroids in HL treatment regimens have not been specifically addressed in (pre) clinical studies. Novel strategies with less toxicity-related side effects that show promising results for cHL treatment are brentuximab vedotin, a CD30-dircted antibody conjugate, and anti-PD1 antibodies nivolumab and pembroluzimab^[24]^.

Non-Hodgkin lymphoma (NHL) is the most frequent hematological cancer in adults and represents a broad collection of more than 30 different subtypes of lymphoid malignancies, both of mature B-cell and T-cell/Natural Killer-cell origin^[25]^. Common types include diffuse large B cell lymphoma (DLBCL), follicular lymphoma, mantle cell lymphoma (MCL) and the more indolent group of marginal zone lymphomas. Despite this heterogeneity, most NHL with progressive disease are treated with relatively similar treatment protocols, which consist of immuno-chemotherapy that includes synthetic GCs^[25]^. This typically involves rituximab in combination with CHOP (cyclophosphamide, doxorubicin, vincristine and prednisone). Other approaches for low to intermediate risk DLBCL comprise of alternative anthracycline-based combinations (R-ACVBP)^[26]^, and for aggressive DLBCL dose-adjusted administration of cytotoxic agents (DA-EPOCH-R), which yields a durable remission in patients with MYC-rearranged B-cell lymphoma^[27]^. For indolent NHL and MCL, the combination of bendamustine plus rituximab has shown improved long-term disease control compared to R-CHOP^[28]^. Most of the *in vitro* studies on differential therapy response in the more common NHL subtypes have addressed chemoresistance to the combined CHOP cocktail, but not directly for the individual drugs. Consequently, specific mechanisms that relate to GC resistance in NHL remain to be identified. In recent years, novel therapies for relapsed/refractory disease in NHL involve CAR T-cell therapy and small molecule inhibitors, such as Bruton’s tyrosine kinase inhibitor ibrutinib, and several phosphatidylinositol 3-kinase (PI3K) and mTOR inhibitors.

Multiple myeloma (MM) is the second most common hematologic malignancy worldwide and two-thirds of the patients diagnosed with MM are above 65 years of age. MM is a heterogeneous clonal plasma cell proliferative disorder, which is almost always preceded by an asymptomatic premalignant stage, termed monoclonal gammopathy of undetermined significance. Diagnosis of MM is based on clinicopathological manifestations of serious end-organ damage, such as osteolytic bone lesions and renal failure^[29]^. MM is still not curable, and autologous SCT remains the standard treatment option for the younger and fit MM patients. As part of the induction therapy before SCT and for MM patients not eligible for SCT, different chemotherapeutic regimens in combination with proteasome inhibitors, immunomodulatory drugs (IMiDs) and GCs have clearly improved the prognosis^[30,31]^. Thus, GCs combined with IMiDs form the cornerstone drugs in many treatment regimens and GC monotherapy induces apoptosis in primary MM cells and in MM cell lines^[32]^.

## Mechanism of action of GCs

**Figure 1:**
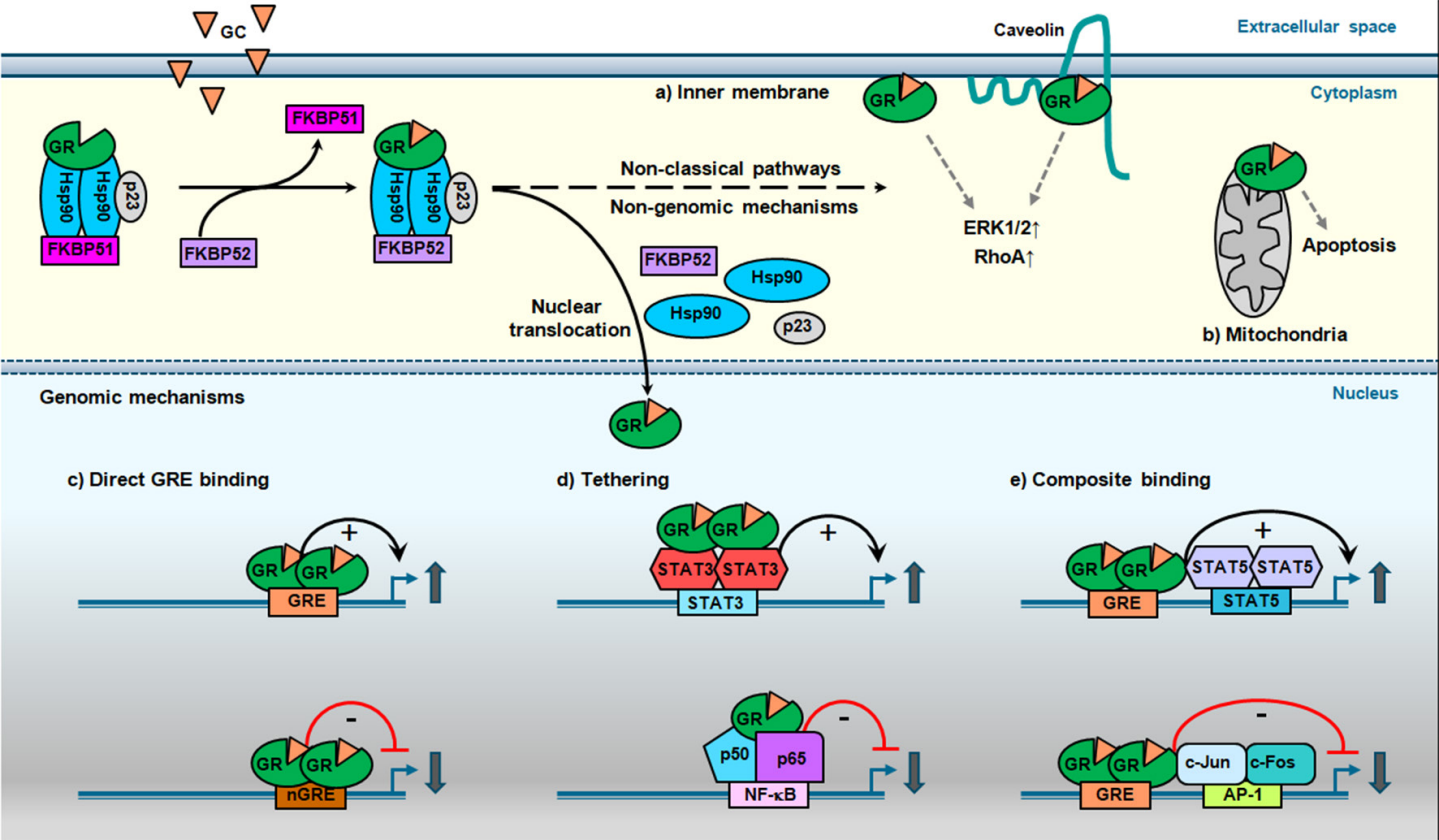
Mechanisms of GR action upon GC stimulation. Upon glucocorticoid (GC) binding, the cytoplasmic glucocorticoid receptor (GR), in complex with accessory proteins Hsp90, p23 and FKBP51, undergoes a conformational change and interacts with FKBP52. This results in dissociation of the multiprotein complex and facilitates subsequent GR signaling. The classical pathway involves genomic mechanisms of gene regulation by GR, while the non-classical pathway results in: a) inner membrane localization of GR, which can lead to ERK1/2 and RhoA activation; b) translocation of GR to mitochondria where it results in inhibition of apoptosis in GC-sensitive cells. Nuclear translocation of GR enhances or represses transcription of target genes by c) direct binding to glucocorticoid responsive element (GRE) or negative GRE (nGRE) sites; d) tethering to other transcription factors without direct GRE interaction; e) or in a composite manner, which involves adjacent GRE binding

The GR is composed of a N-terminal transactivation domain, a central DNA-binding domain and a C-terminal ligand-binding domain^[33,34]^. The DNA-binding domain contains two zinc-finger motifs that recognize and bind specific target DNA sequences, termed glucocorticoid- responsive elements (GREs)^[35,36]^. In the absence of GCs, the GR is primarily present in the cytoplasm as part of a larger multiprotein complex that includes the chaperone protein HSP90, p23 and immunophilin-related co-chaperone FKBP51^[37,38]^, which keep GR in an inactive state. Binding of GCs yields a conformational change in the GR with rearrangement of the multiprotein complex, exposure of the nuclear localization signals and nuclear import^[39]^. Within the nucleus, the receptor dimerizes and binds to GREs to stimulate expression of target genes [Figure 1]. These conventional GREs belong to a family of imperfect palindromes consisting of two inverted hexameric half-site motifs (AGAACA) separated by 3 base pairs, which facilitate GR dimerization on the element^[36]^. Alternatively, GR associates with so-called negative GREs (nGREs) to repress gene transcription^[40,41]^. These elements contain the consensus sequence CTCC(n)_0-2_GGAGA that differs dramatically from the activating sequences. Upon DNA binding, conformational changes within GR lead to the recruitment of coregulators and chromatin-remodeling complexes that influence the activity of RNA polymerase II. Both the type of ligand and the GRE sequence itself can dictate the specific assembly and function of cofactors depending on the alterations induced in the receptor structure^[42,43]^. Interestingly, certain noncoding RNA fragments, such as Gas5, may act as decoy GRE, thus competing with DNA GREs for binding to GR^[44]^.

GR is also able to regulate gene transcription through physical association with other transcription factors, which can completely rely on GR tethering to these DNA-bound proteins or involve GR binding to both a GRE and the transcription factor on an adjacent site in a composite manner. The interaction of GR with the transcription factors NF-κB (GR tethering) and AP-1 (composite binding) inhibits their activity^[45-49]^, which accounts largely for the anti-inflammatory and immune-suppressive effects of GCs. This negative regulation by GR tethering (also together with SMAD3, POU2F1 and T-bet) and composite binding (including also C/EBPβ, GATA1) is known as transrepression and is believed to involve the activity of GR monomers^[50]^. In contrast to these inhibitory effects, the interaction of GR with STAT3 and STAT5 transcription factors, either apart from or in conjunction with GRE binding, may enhance transcriptional activation of certain target genes^[51-53]^.

Finally, GR can sometimes undergo non-canonical activation and can be transcriptionally active in the absence of ligand, or mediate transcription-independent effects even in the presence of ligand^[54,55]^. These rapid non-classical GR effects relate to the presence of GR on the inner cell surface and stimulation of RhoA signaling^[56]^, or through direct activation of extracellular signal regulated kinase-1/2 (ERK1/2)^[57,58]^. In fact, cooperation between non-classical and classical GC signaling has been described, where the slower acting classical GC signaling mediates many of the physiological important responses^[58]^. Additional non-genomic mechanism includes translocation of GR into mitochondria specifically in lymphoid cells, which has been shown to correlate with GC-mediated apoptosis in thymocytes and absent in GC-resistant T cell lymphomas^[59,60]^.

## Alterations in GR expression and function contribute to GC resistance

The human NR3C1 gene is located on chromosome 5q31-32 and composed of nine exons. Functionally distinct GR isoforms can be generated by alternative splicing and translation initiation^[61]^. Alternative splicing that occurs at the 3’ end of the NR3C1 gene generates the receptor isoforms GRα and GRβ, which differ at their extreme C-termini. The GRα protein encodes the prototypic functionally active receptor, while the splice variant GRβ utilizes an alternative splice acceptor site in exon 9, yielding a shorter protein with a distinct 15-amino acid stretch at the C terminus. This unique GRβ sequence abrogates ligand binding, and yields a constitutive nuclear isoform that has been reported to act as a dominant-negative inhibitor of GRα on genes both positively and negatively regulated by GCs^[62,63]^, although this has been disputed by others^[64-66]^. In fact, subsequent studies demonstrated that GRβ can directly induce and repress gene expression independent GRα transcriptional activity^[67]^. Thus, by its ability to regulate gene expression, alterations in the expression level of GRβ may modulate the cellular sensitivity to GCs^[68,69]^. Increased levels of GRβ have been observed in a single case of GC-resistant CLL^[70]^, and lower GRα:GRβ mRNA expression ratios correlate with reduced sensitivity towards GC-induced apoptosis in childhood ALL^[71]^.

Besides GRβ, several additional GR isoforms arise due to alternative splicing of the NR3C1 gene that can impact GC signaling. GRγ originates from the use of an alternative splice donor site in the intron separating exon 3 and 4, which yields an insertion of a single arginine residue between the two zinc-fingers of the DNA-binding domain^[72]^. This widely expressed GRγ isoform binds GCs and DNA with a similar capacity as GRα, but its ability to activate GC-responsive reporters is compromised, and GRγ exhibits a distinct transcriptional profile compared to GRα^[36]^. The role of GRγ in mediating GC therapy resistance in lymphoid malignancies has not been firmly established. Two studies reported lower steady-state and GC-induced GRγ mRNA levels in ALL leukemic blasts of prednisone good responders (PGR) compared to prednisone poor responders (PPR)^[73,74]^, while others showed that GRγ mRNA induction *in vivo* in childhood ALL patients was more rapid and higher in PGR compared to PPR^[75]^. Two additional splice variants exist that lack large regions of the ligand-binding domain, termed GR-A and GR-P. Little is known about GR-A, but GR-P appears to be a predominant receptor variant in lymphoid malignancies, including MM^[76,77]^, but not exclusively in GC-refractory cases.

**Figure 2:**
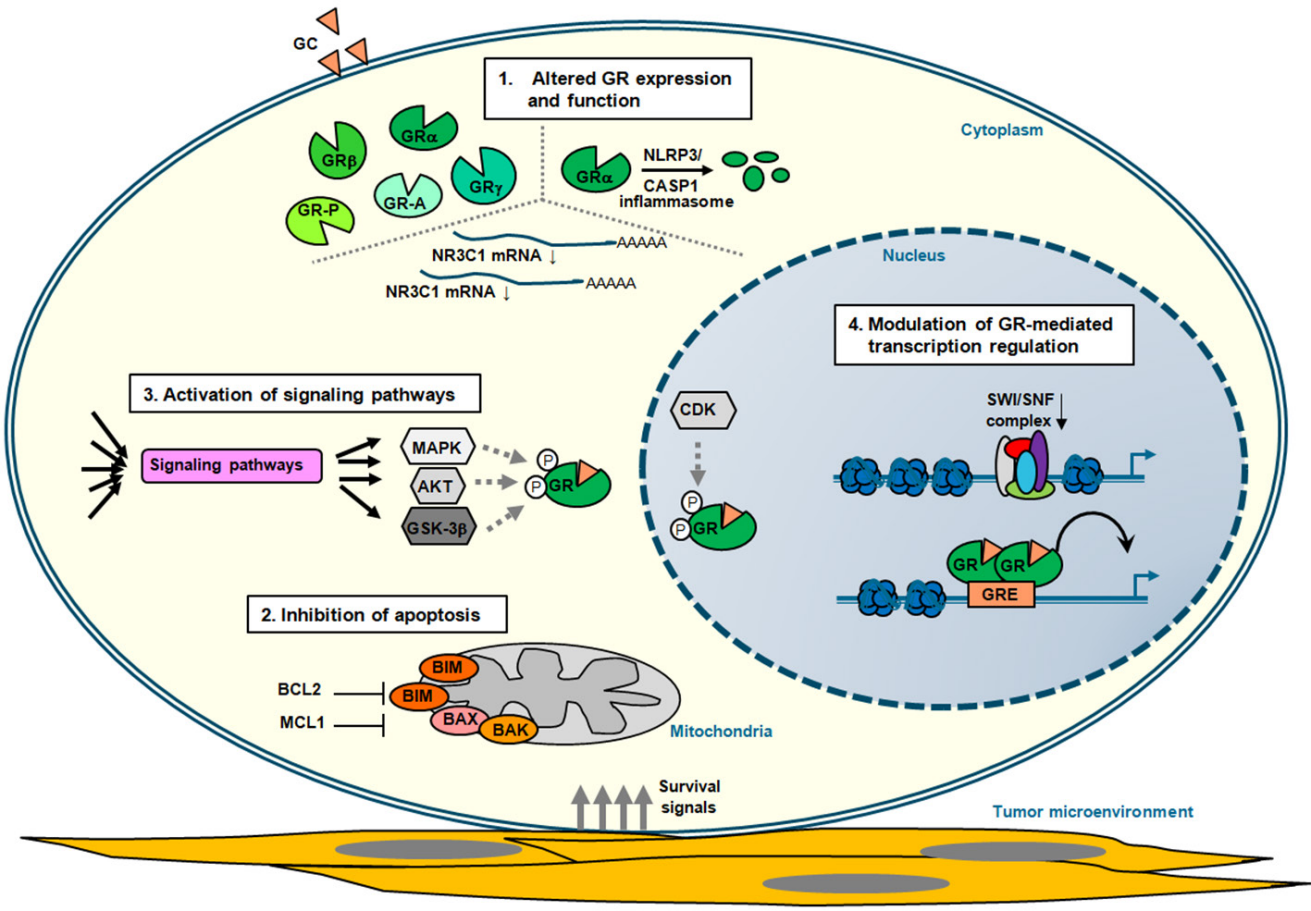
Mechanisms of GC resistance in lymphoid malignancies. Schematic representation of the four main mechanisms that contribute to glucocorticoid (GC) resistance in lymphoid tumor cells (see also Table 1). These represent altered: glucocorticoid receptor (GR) expression due to alternative splicing and translation initiation (see text) and function; inhibition of apoptosis by deregulated expression of proteins involved in programmed cell death and through interaction with components of the tumor microenvironment that promote survival signaling; activation of signaling pathways that in a direct or indirect manner alter GR-mediated cell death (see text); and modulation of GR-mediated transcription regulation, thereby altering the expression of critical regulators of GC therapy sensitivity

In a recent study, none of the alternative splicing variants showed a correlation with GC sensitivity in BCP-ALL, but only GRα played a central role in GC-mediated pro-apoptotic activity^[78]^. Thus, it seems that the relative abundance of GRα in comparison to the other isoforms is one of the more relevant determinants for GC responses. Acquired GC resistance in cancer patients may relate to inter-individual differences in the functional GRα pool before receiving therapy or reduced upon GC treatment. In fact, GC-dependent regulation of the *NR3C1* gene is one of the critical determinants of GC sensitivity and resistance^[79-81]^. Thus, while most tissues show negative auto-regulation of *NR3C1*, the promoter is positively regulated in GC-sensitive lymphoid cells. Both promoter/enhancer DNA elements-dependent and -independent mechanisms have been proposed to be involved in the repression of GRα mRNA expression^[82-84]^. Next to altered mRNA expression, a block in transcription elongation of *NR3C1* has been linked to GC resistance in MM^[85]^. In BCP-ALL, focal *NR3C1* deletions and inactivating mutations have been identified that contribute to a diminished pool of functional GRα^[86,87]^, which are often relapse-associated^[88,89]^. Furthermore, microRNAs miR-130b, miR-142-3p and miR-124 targeting 3’UTR of GRα in MM and ALL, promote GC resistance by downregulation of GRα mRNA transcripts^[90-92]^. In addition, proteasome-mediated degradation of the GRα protein results in increased turnover of the receptor^[93]^. Another pathway that regulates GR levels is the NLRP3-CASP1 inflammasome, and increased levels of *NLRP3* and *CASP1* expression in GC-resistant and relapsed ALL samples due to promoter hypomethylation of these genes promote GR cleavage and GC resistance [Figure 2 and Table 1]^[94]^.

**Table 1:** Mechanisms contributing to glucocorticoid resistance in lymphoid malignancies

Type of mechanism	Specific examples	Ref.
Altered GR expression and function	Differential expression of alternatively spliced and translated GR isoforms	[68-71,73-78,97,98]
	Transcriptional autoregulation of *NR3C1*	[80,81,85]
	*NR3C1* gene deletions and mutations	[86-89]
	Regulation of GR by microRNAs	[90-92]
	Degradation of GR by NLRP3-CASP1 inflammasome	[94]
Inhibition of apoptosis	*BCL2* translocation	[102]
	Differential BIM expression	[105-121]
	Altered MCL1 expression	[122-127]
	Loss-of-function mutations in PRC2 complex proteins (EZH2, EED, SUZ12)	[128]
	Survival signals by stromal cells	[130,131]
Activation of signaling pathways	MAPK pathway	[140]
	CDK-dependent GR phosphorylation	[146,147]
	PI3K/AKT pathway	[148-153]
	LCK signaling	[156]
	JAK/STAT pathway	[160]
	Metabolism/mTOR signaling	[161-163]
	NOTCH1 pathway	[165-167]
Modulation of GR-mediated transcription regulation	Reduced expression of SWI/SNF chromatin remodeling complex proteins	[122,171]
	*IKZF1*, *BTG1*, *TBL1XR1* and *CREBBP* gene deletions/mutations affect GR target gene regulation	[179-187]
	Chromatin accessibility *BIM* locus	[190]

GR: glucocorticoid receptor; MCL: mantle cell lymphoma; PI3K: phosphatidylinositol 3-kinase; CDK: cyclin-dependent kinase

An additional mechanism that contributes to even a larger diversity of GR proteins results from alternative translation initiation of the GR mRNA transcripts^[95,96]^. From the classic full-length GRα isoform, eight proteins can be generated due to alternative AUG start codons with a progressively shorter N-terminal transactivation domain (GRα-A, GRα-B, GRα-C1, GRα-C2, GRα-C3, GRα-D1, GRα-D2 and GRα-D3)^[61]^. Each of the other GR splice variants (GRβ, GRγ, GR-A and GR-P) are also expected to give rise to similar translational isoforms. For the GRα translational isoforms, no significant differences have been observed in ligand affinity or their capacity to bind to GREs following GC stimulation^[97]^. However, the subcellular localization of the translational isoforms is different, with GRα-D residing constitutively within the cell nucleus, while the other isoforms are predominantly cytoplasmic in the absence of ligands^[95]^. Notably, cells expressing GRα-C are most sensitive to GC-induced cell killing, whereas cells expressing GRα-D are the most resistant^[97,98]^. These phenotypes correlate with the gene regulatory profiles, where the GRα-C isoform is the most active in the ability to enhance transcription of GC-responsive reporter and endogenous genes^[95,97]^.

## GC resistance by intrinsic inhibition of apoptosis

The process of programmed cell death or apoptosis is characterized by the activity of caspases that can be activated by two main pathways^[99]^. The intrinsic mitochondrial pathway relies on mitochondrial outer membrane permeabilization and release of proteins from the mitochondrial intermembrane space. Among these, cytochrome C associates with Apaf-1 and the initiator caspase-9, which leads to activation of effector caspase-3 and caspase-7. The BCL2 family act as central regulators of mitochondrial outer membrane permeabilization leading to the irreversible release of intermembrane space proteins, subsequent caspase activation and apoptosis^[100]^. The BCL2 family is divided into three groups based on their primary function: (1) anti-apoptotic proteins (BCL2, BCL-X_L_, BCL-W, MCL1, BFL1/A1); (2) pro-apoptotic pore-formers (BAX, BAK, BOK); and (3) pro-apoptotic BH3-only proteins (BAD, BID, BIK, BIM, BMF, HRK, NOXA, and PUMA). All BCL2 family proteins contain a BH3 domain, which represents one out of four BH domains involved in interactions between these proteins. Both the anti-apoptotic and pore-forming proteins contain all four BH domains (multi-BH domain proteins), while the BH3-only proteins are subdivided into activator (BIM, BID, PUMA) and sensitizer (BAD, NOXA, BIK, BMF, HRK) proteins^[101]^. The second apoptosis pathway is the extrinsic pathway, which is dominated by signaling through death ligands, as FasL and TNF, and is not directly involved in GC-induced apoptosis.

In several subtypes of NHL, BCL2 protein expression is increased due to t(14;18)(q32;q21) translocations involving the *BCL2* gene. DLBCL positive for both c-*MYC* and *BCL2* translocations, called double-hit lymphoma, display inferior outcome after R-CHOP treatment^[102]^, probably by suppressing in part GC-induced apoptosis in these lymphoma cells. Transcriptional induction of the pro-apoptotic BH3-only protein BIM by GCs has been observed in many lymphoid cell types^[103]^, and is considered to be one of the key mediators for the selective pro-apoptotic response in lymphoid cells to GCs. However, the mere up-regulation of the pro-apoptotic BIM protein is insufficient for initiating apoptosis, and requires activation through post-translational modifications and interactions with other proteins^[104]^. BIM is involved in GC-induced cell killing in ALL^[105-110]^, CLL^[111,112]^, and MM^[113-116]^. FOXO3a is a critical regulator of *BIM* expression^[117,118]^, and inhibition of the FOXO3a/BIM axis is observed in GC-resistant T-ALL^[119]^, but also epigenetic silencing of *BIM*(*BCL2L11*) expression is detected in pediatric ALL poor prednisone responders^[120]^. Opposing mechanisms of *BIM* and *BCL2* gene regulation in dexamethasone-sensitive and -resistant pediatric ALL xenografts correlates with differential response towards GC-induced apoptosis^[121]^. In addition, increased levels of anti-apoptotic protein MCL1 correlate with GC resistance in ALL^[122-125]^. *MCL1* and pro-apoptotic gene *NOXA* are direct targets of GR and modulate the response to GCs^[126]^. In plasma cell malignancies MM and Waldenström macroglobulinemia, MCL1 and BCL2 are associated with decreased sensitivity towards GCs^[127]^.

Besides aberrant expression of proteins directly involved in programmed cell death, there are also genetic alterations that can mediate therapy resistance by altering the expression of central regulators of the mitochondrial apoptosis process [Table 1]. Loss-of-function mutations of polycomb repressive complex (PRC2), which includes *EZH2*, *EED* and *SUZ12*, inhibit mitochondrial apoptosis in immature T lymphocytes, in part through upregulation of HSP90 family chaperone tumor necrosis factor receptor-associated protein 1 (TRAP1)^[128]^. TRAP1 regulates a variety of cellular processes, and is involved in the protection against DNA damage and apoptosis induced by oxidants and other forms of cellular stress^[129]^. Consequently, loss of PRC2 function in mouse T cell progenitors and T-ALL cells results in mitochondrial apoptosis resistance towards several cytotoxic chemotherapeutics, including dexamethasone^[128]^. In addition, localization of malignant lymphoid cells within the bone marrow (BM) niche provides survival signals via cytokines and growth factors, as well as interaction with specific components in the tumor microenvironment, which protects cells from GC-induced apoptosis. For instance, epithelial membrane protein 1 (EMP1) has been directly linked to inferior prognosis in pediatric ALL, and EMP1 contributes to GC resistance mediated through adhesion to mesenchymal stromal cells^[130]^. Also for MM, protective effects of pro-survival cytokines from the BM microenvironment confer therapy resistance^[131]^.

## Signaling pathways that impact GC resistance

GRα is subject to various post-translational modifications, which include phosphorylation, sumoylation, acetylation and ubiquitination^[61]^. Studies have mainly linked alterations in GRα phosphorylation status to GC resistance, although sumoylation seems also to play an important role in regulating GR activity^[132]^. Ligand-dependent GR phosphorylation significantly affects the cellular response to steroids by its ability to modulate the cellular trafficking of the receptor, its protein stability, and transcriptional activity^[133]^. Several kinases have been shown to phosphorylate GRα primarily at the N-terminal transactivation domain, which include mitogen activated protein kinases (MAPKs), cyclin-dependent kinases (CDKs), AKT/protein kinase B as well as glycogen synthase kinase 3 beta (GSK-3β) and alpha (GSKα)^[134]^.

MAPKs constitute the largest subfamily of serine/threonine kinases that transduce extracellular signals into intracellular processes and altered gene expression^[135]^. In the context of GR signaling, p38 MAPK, c-Jun N-terminal kinase (JNK) and ERK have been identified as regulators of GR function. p38 MAPK-mediated phosphorylation of GR on serine residue 211 (Ser211) enhances its target gene regulation and facilitates GC-induced apoptosis^[136]^, while JNK phosphorylates GR on Ser226 suppressing its transcriptional activity and blocking apoptosis^[137,138]^. Furthermore, p38 MAPK phosphorylates GR on Ser134 in response to stress, resulting in increased association with 14-3-3 proteins and selective target gene regulation^[139]^. Inhibition of the more upstream MAPK pathway members MEK2 and MEK4 increases the sensitivity to prednisolone in pediatric ALL, which seems to correlate with increased phospho-ERK levels and GC resistance at relapse^[140]^. ERK inhibition reduces Ser203 phosphorylation resulting in enhanced GR nuclear localization and GC-mediated target gene regulation^[141]^.

CDK proteins in complex with cyclins act as key regulators of the cell cycle, and several CDK complexes (i.e., cyclin A-CDK2, cyclin B-CDK2 and cyclin E-CDK2) phosphorylate GR on serine residues 203 and 211 (Ser203 and Ser211)^[133,142]^. CDK5 has no cell cycle regulatory function but is activated upon stress, and CDK5 phosphorylates GR on multiple serine residues (Ser203, Ser211 and Ser226)^[143]^. GR phosphorylated on Ser203 does not localize to the nucleus and is transcriptionally inactive^[144]^. GR phosphorylated on Ser211 displays the highest transcriptional activity and recruitment to GRE-containing promoters, while Ser226 is associated with a negative effect on transcription regulation^[144,145]^. In fact, lack of Ser211 phosphorylation has been directly linked to GC resistance in lymphoid cells^[146]^, and the combined ratio of activating Ser211 and inhibitory Ser226 phosphorylation of GR in CLL predicts GC-responses^[147]^.

The PI3K/AKT pathway plays a central role in regulating cell growth and survival of lymphoid cells downstream of B- (BCR) and T-cell receptor (TCR) signaling as well as cytokine receptor stimulation. Constitutive PI3K activation is frequently observed in lymphoid malignancies, including ALL, CLL and MCL^[148,149]^, which can be attributed to loss of PTEN function, or mutations that mimic BCR signaling. Pharmacologic inhibition of PI3K or AKT can overcome GC resistance in ALL^[150-152]^, which has been directly linked to inhibition of AKT1-mediated phosphorylation of GRα on Ser134^[152]^. This results in cytoplasmic retention of GRα via interaction with 14-3-3 proteins, although AKT1 has also a positive effect on GR transcriptional activity within the nucleus^[152,153]^. Notably, recent studies show that PI3K/AKT/mTOR pathway also affect GR phosphorylation at Ser211, inhibiting GRα nuclear import and shift GRα transcriptional activity towards gene transrepression in both lymphoid and epithelial cells^[154,155]^.

Activation of the PI3K pathway may also result through autocrine and paracrine release from cytokines in lymphoid tumors, and LCK hyperactivation in GC-resistant T-ALL results in upregulation of calcineurin/nuclear factor of activated T cells signaling, which triggers interleukin-4 (IL-4) overexpression^[156]^. LCK inhibition can revert this GC resistance and induce cell death in T-ALL cells insensitive for GC treatment. To the converse, IL-4 stimulation alone is sufficient to impose GC resistance in dexamethasone-sensitive T-ALL cell lines^[156]^, most likely through activation of the PI3K/AKT pathway. GSK3β, known to participate in immunity and metabolism regulation, phosphorylates GR on Ser404 upon GC treatment, thereby adjusting the repressive effects of GR on NF-κB^[157]^. However, GSK3β has also a strong pro-apoptotic function and is negatively regulated by the PI3K/AKT pathway. In part, this relates to destabilization of MCL1 through GSK3β-mediated phosphorylation^[158]^, thereby influencing the sensitivity towards GC-induced apoptosis in an indirect manner. GSK3α on its turn interacts with GRα in the absence of ligand. Upon exposure to GCs, GSK3α dissociates from GRα and interacts with pro-apoptotic protein BIM, and this interaction is also observed for GSK3β^[159]^. Thus, inhibition of GSK activity contributes to GC resistance at different levels.

Other pathways that impact GC responses in lymphoid malignancies are JAK/STAT, mTOR and NOTCH1 signaling. Activation of the JAK/STAT pathway is commonly observed both in ALL and mature lymphoid malignancies. Inhibition of JAK/STAT signaling in T-ALL overcomes IL-7-induced GC resistance in a subset of T-ALL^[160]^. Interestingly, in ALL, GC resistance has been directly associated with the metabolic state of these cells, including upregulation of glycolysis, oxidative phosphorylation and activation of mTOR signaling^[161,162]^. Inhibition of mTOR by rapamycin, blocks upregulation of MCL1, thereby facilitating the pro-apoptotic action of BIM^[123]^. However, it has been argued that the glycolytic reserves are relative low in BCP-ALL, resulting in the constitutive activation of the LKB1-AMPK energy-stress-sensor-pathway^[163]^. B-lymphoid transcription factors are responsible for this metabolic state and set the threshold for GC responses, in part by positively regulating *NR3C1* levels^[163]^. On the other hand, it has been reported that suppression of B cell development genes is required for GC-induced cytotoxicity^[164]^. In T-ALL, NOTCH1 activation is frequently observed, which has been shown to impair GC signaling^[165]^. Combined expression of NOTCH1 and BCL2 results in GC-resistant T cell lymphoma^[166]^. In GC-resistant T-ALL, abrogation of active NOTCH1 signaling by gamma-secretase inhibitors reverses GC resistance^[165]^. Notably, NOTCH1 target gene HES1 acts as a negative regulator of GR-mediated gene transcription through binding to adjacent N-boxes (CACNAG) in a composite manner, and GC signaling requires silencing of *HES1* expression^[167]^.

## GC resistance through modulation of GR-dependent transcription regulation

GC-induced RNA transcription within the nucleus heavily depends on specific protein-protein interactions and chromatin context, which together control GR-mediated gene regulation^[168]^. Binding of agonist-liganded GR to conventional GREs confers transcriptional transactivation through association with co-activators, like SRC1, TIF2/SRC2 and SRC3^[169]^, as well as CBP/p300^[157]^. GR can also recruit BRG1, the central ATPase of the SWI/SNF chromatin remodeling complex, which facilitates the formation of transcriptional pre-initiation complex and transcriptional transactivation^[170]^. Several of the core subunits of the SWI/SNF complex, i.e., SMARC4A, SMARCB1 and ARID1A, are lower expressed in GC-resistant ALL^[122,171]^. Coactivator function of GR on selective target genes can also involve association with Mediator subunits MED1 and MED14^[172,173]^. Transcriptional suppression of GR occurs via transrepression of AP-1/NF-κB sites^[168,174]^, blocking chromatin accessibility of AP-1 and NF-κB through activation of powerful negative regulators of the inflammatory signaling cascade^[175]^, or direct GR association with nGREs^[40,176]^. Negative enhancers selective facilitate GR recruitment of the co-repressor GRIP1^[176,177]^. Furthermore, negative autoregulation of *NR3C1* expression by GC is mediated by NCoR repression complex through long-range chromatin interactions with a nGRE in exon 6 of *NR3C1*^[83]^. On the other hand, histone H3 lysine-9 methyltransferase G9a/KMT1C mediates both coactivator and corepressor function for hormone-activated GR^[178]^.

Attenuation of GR-mediated transcription regulation both at the level of GC-induced gene activation and repression has been observed in *IKZF1*-deleted BCP-ALL, which is associated with inferior treatment outcome, relapsed disease and reduced GC-sensitivity^[179-181]^. Focal *BTG1* deletions further enhance this phenotype^[182]^, which correlates with the ability of BTG1 to regulate GR-dependent transcription^[183]^. *TBL1XR1* gene deletions are enriched in relapsed BCP-ALL and *TBL1XR1* knockdown confers GC resistance in BCP-ALL cell lines^[184]^. TBL1XR1 is a member of the NCoR complex and responsible for NCoR degradation^[185,186]^. TBL1XR1 silencing yields decreased GR recruitment at gene regulatory regions, which is accompanied by increased NCoR1 residing on the promoters of GC-responsive genes with increased recruitment of HDAC3^[184]^. In addition, inactivating mutations in *CREBBP*, which encodes the important GR transcriptional coactivator CBP, have been detected in relapsed ALL, and impair GC-induced transcriptional responses^[187]^.

Next to regulation at the level of GR activity itself, selective differences in chromatin structure also pre-determine effective GR binding and ensure appropriate transcription regulation^[188,189]^. Notably, lymphocyte-specific chromatin accessibility has been linked to acquired GC resistance in ALL for individual patients that display reduced sensitivity to GCs as well as for non-lymphoid cell types that are intrinsically resistant to GC-induced apoptosis^[190]^. This study revealed that the selective induction of pro-apoptotic gene *BIM*(*BCL2L11*) in GC-sensitive ALL samples correlates with a defined open and active chromatin structure at an intronic GR-binding region within the *BIM* locus together with recruitment of the chromatin architectural protein CTCF to this site. Reduced accessibility of the intronic *BIM* locus in GC-resistant ALL samples was associated with increased levels of DNA methylation and histone acetylation, which could be reversed by DNA demethylating drug azacitidine and histone deacetylase (HDAC) inhibitor vorinostat, respectively^[120,190]^.

## Conclusion and perspective

In recent years, a deeper understanding on the mechanism of action of GC signaling in normal and malignant lymphoid cells has provided valuable insight into the pathways that regulate GR function and the selectivity of target gene regulation. Many of these physiological processes dictate the sensitivity and specificity of GC responses. To the converse, functional studies linked to genetic aberrations or altered expression signatures associated with inferior prognosis and GC resistance in different lymphoid malignancies have also contributed to a better understanding of GR regulation [Table 1]. Thus, acquired GC often results from the formation of highly resistant leukemia or lymphoma-associated subclones that arose even in the absence of GC-induced selection pressure, but provided selective growth advantage during the development of the tumor. In many cases, the genetic aberrations present in these subclones are maintained or enriched at time of relapse, since they impose specific survival cues during treatment by conferring partial resistance to GC-induced apoptosis. For some lymphoid malignancies, such as ALL, many of these relapse-associated gene mutations or copy number abnormalities have been identified, and functional studies are underway to address the exact mechanisms underlying GC therapy resistance for each of the affected genes and pathways. However, there are still clinically relevant biomarkers associated with inferior prognosis and GC resistance in other lymphoid malignancies that remain to be identified. In the near future, most of these relapse-enriched or relapse-specific genomic aberrations will be identified by employing whole exome sequencing and transcriptome analyses in the different types of lymphoid malignancies.

The challenges for the near future to improve treatment outcome in high-risk patients and for hematological cancers that are still incurable is to target the mechanisms that impose therapy resistance and provide precision medicine that is tailored to the individual (epi-)genetic and immunological context of each tumor. Therapies breaking GC resistance will be beneficial for those treatment regimens that are relatively more dependent on the cytotoxic activity of synthetic GCs, as is probably the case in ALL and MM. The development of different targeted small molecule inhibitors and modalities of immunotherapy in combination with predictive biomarkers for personalized treatment will hopefully increase the overall survival rate of hematological cancer patients.

## Declarations

### Copyright

© The Author(s) 2019.
